# Functional connectivity of motor cortical network in patients with brachial plexus avulsion injury after contralateral cervical nerve transfer: a resting-state fMRI study

**DOI:** 10.1007/s00234-017-1796-0

**Published:** 2017-02-24

**Authors:** Aihong Yu, Shufeng Wang, Xiaoguang Cheng, Wei Liang, Rongjie Bai, Yunhao Xue, Wenjun Li

**Affiliations:** 1 Department of Radiology, Beijing Jishuitan Hospital, The 4th Medical College of Peking University, 31 Xinjiekou E Rd, Xicheng Qu, Beijing, 100035 China; 2 Department of Hand Surgery, Beijing Jishuitan Hospital, The 4th Medical College of Peking University, Beijing, China

**Keywords:** Contralateral C7 nerve transfer, Brachial plexus avulsion injury, Functional connectivity, Resting state, Cerebral plasticity

## Abstract

**Introduction:**

The purpose of this study is to assess the functional connectivity of the motor cortical network in patients with brachial plexus avulsion injury (BPAI) after contralateral C7 nerve transfer, using resting-state functional magnetic resonance imaging (RS-fMRI).

**Methods:**

Twelve patients with total brachial plexus root avulsion underwent RS-fMRI after contralateral C7 nerve transfer. Seventeen healthy volunteers were also included in this fMRI study as controls. The hand motor seed regions were defined as region of interests in the bilateral hemispheres. The seed-based functional connectivity was calculated in all the subjects. Differences in functional connectivity of the motor cortical network between patients and healthy controls were compared.

**Results:**

The inter-hemispheric functional connectivity of the M1 areas was increased in patients with BPAI compared with the controls. The inter-hemispheric functional connectivity between the supplementary motor areas was reduced bilaterally.

**Conclusions:**

The resting-state inter-hemispheric functional connectivity of the bilateral M1 areas is altered in patients after contralateral C7 nerve transfer, suggesting a functional reorganization of cerebral cortex.

**Electronic supplementary material:**

The online version of this article (doi:10.1007/s00234-017-1796-0) contains supplementary material, which is available to authorized users.

## Introduction

Brachial plexus avulsion injury (BPAI) is the most severe peripheral nerve injury and typically results in paralysis of the affected upper limb, which significantly reduces quality of life. Surgical restoration of hand function, especially finger flexion, is challenging in patients with total BPAI. Contralateral C7 nerve transfer to the median nerve via a long vascularized ulnar nerve graft is an effective procedure that was first developed by Gu et al. in clinical studies [1–4]. After contralateral C7 nerve transfer, the bilateral limbs of patients with BPAI share a single pyramidal pathway, descending from the cerebral cortex ipsilateral to the affected plexus, resulting in restoration of the affected arm function.

In early post-operative stages, the motor function recovery appeared only as bilateral associated movements. However, a few patients regained independent motor function of the affected wrist and fingers following long-term functional exercise [5]. The unique clinical phenomenon suggests extensive cortical reorganization in the bilateral motor cortex. However, the central mechanisms underlying motor plasticity after contralateral C7 nerve transfer remain unclear, although a few functional imaging studies showed intra-hemispheric and inter-hemispheric cortical plasticity in rats and humans [6–10]. However, the interactions of motor networks in the resting state after contralateral C7 nerve transfer have not yet been investigated. Resting-state functional magnetic resonance imaging (RS-fMRI) has been used to explore the inter-regional correlation in terms of functional connectivity or “intrinsic connectivity,” which is thought to reflect the intrinsic functional architecture of the human brain [11–13]. This technique has been widely used to investigate functional reorganization in subjects suffering from central or peripheral nervous system disease [14–16].

In the present study, the functional connectivity of the motor cortical network in patients with BPAI after contralateral C7 nerve transfer has been assessed using RS-fMRI.

## Material and methods

### Subjects

The subjects included 12 patients with unilateral total BPAI due to traffic accidents (7 men and 5 women, mean age 26.2 ± 9.1 years, range 17–54 years). On average, the interval between trauma and contralateral C7 transfer surgery was 3.5 months, ranging from 1 to 13 months. The average interval between contralateral C7 transfer surgery and functional MRI was 34.4 months, ranging from 18 to 48 months (Table 1). All the patients had complete avulsion of the five roots, and the muscle strength of finger and wrist flexion in all the patients was graded as M0 before contralateral C7 nerve transfer. The preliminary diagnosis of complete brachial plexus injury was based on detailed history, meticulous physical examination, and results using needle electromyography (EMG). All the patients were subjected to computed tomographic myelography (CTM) and MRI of brachial plexus. MRI of brachial plexus was acquired at two centers randomly to reduce travel time of the patients in the study. Acquisition parameters were adjusted to be as equal as possible between the two scanners at two centers, while still having near optimal settings for each system. Each patient underwent clinical evaluation for residual motor function and signs of associated neurological lesions. All the patients underwent surgical exploration and contralateral C7 nerve transfer via the modified pre-spinal route and direct coaptation of the contralateral C7 nerve with the lower trunk to restore finger flexion [3]. All the surgical interventions were performed by the same medical team at Beijing Jishuitan Hospital. The modified British Medical Research Council (MRC) muscle grading system [4] was used to evaluate the motor function after the patients regained finger flexion. The strength of finger flexion was tested with the wrist extended 20° to 30°. Muscle strength was graded as poor (M0 to M2), fair (M2+ to M3), good (M3+ or M4−), or excellent (M4 to M5−). The muscle strength of finger and wrist flexion in all the patients was graded as M4.

**Table 1:** Patient demographics

Case no.	Sex	Age (years)	Handedness	Lesions	Muscle grade	Time course of fMRI post-surgery (months)
1	F	24	R	R	M4	48
2	F	23	R	L	M4	48
3	M	23	R	R	M4	24
4	M	26	R	L	M4	48
5	M	17	R	R	M4	36
6	M	23	R	R	M4	42
7	F	29	R	L	M4	24
8	M	39	R	R	M4	18
9	F	22	R	L	M4	36
10	F	54	R	R	M4	27
11	M	17	R	L	M4	34
12	M	24	R	L	M4	28

No history of psychiatric or neurological abnormalities was found in any patient. The control group consisted of 17 age-matched healthy volunteers (mean age 26.3 ± 2.8 years, range 21–30 years) with no history of psychiatric or neurological abnormalities. All the participants were right-handed. Handedness was determined using Peking University Hand Preference Inventory. Written informed consent was obtained from all subjects. The study protocol, amendments, and informed consent were reviewed and approved by the local institutional review boards.

#### MR data acquisition

MR imaging was performed using a 1.5-T MR scanner (Siemens Magnetom Espree, Germany). All the data were acquired using a standard quadrature birdcage head coil for both RF transmission and reception. Anatomical images were obtained from axial multi-slice SE T1-weighted images [TR (repetition time) = 500 ms, TE (echo time) = 7.7 ms, matrix size 256 × 256, FOV 230 × 230 mm, slice thickness 5 mm and gap 1.5 mm, 20 slices]. Resting-state functional images were acquired using a whole-brain 3D echo-planar imaging (EPI) sequence [TR 2730 ms, TE 45 ms, flip angle = 90°, matrix 64 × 64, slice thickness 5 mm, gap 1.5 mm, FOV 230 × 230 mm, resolution 3.6 × 3.6 mm in-plane], providing blood oxygenation level-dependent (BOLD) contrast. The resting run generated 180 whole-brain volumes. High-resolution, T1-weighted gradient echo 3D images (magnetization prepared rapid gradient echo imaging (MPRAGE)) were then acquired for coregistration [TR/TE = 1970 ms/3.6 ms, flip angle = 15°, slice plane sagittal, slice thickness = 1 mm, gap 0.5 mm, FOV = 256 × 256 mm, matrix size = 192 × 256, 176 slices].

During the resting fMRI session, the subjects were instructed to keep their eyes closed, to remain motionless, and not to think of anything in particular.

#### Data preprocessing

Resting-state fMRI data were pre-processed using previously described procedures [17]. The following steps were performed: slice timing and rigid body correction for head motion, data smoothing (6-mm full width at half maximum), low-pass temporal filtering, ventricular and white matter signal regression, normalization for global mean signal intensity across runs, and transformation of the data into a standard atlas space. All images were temporarily low-pass filtered (0.01 Hz < frequency < 0.08 Hz).

Whole-brain signal regression was also included in the processing stream, which improved the correction of motion-related artifacts [18]. All subjects included in this study met the quality control criterion of slice-based temporal signal-to-noise ratio >100.

The strength of functional correlation between the motor cortex (M1) and supplementary motor area (SMA) was quantified for each patient using hand motor seed regions (Talairach-Tournoux coordinates ±41, 17, 58) defined from an independent study of actual hand movements [19]. The time course of each region of interest (ROI; radius = 6 mm) was then extracted before functional connectivity analysis. After the ROIs were defined, Pearson’s correlation coefficients between the time course of each ROI and that of every voxel in the whole brain (voxel-wise analysis) were calculated.

Correlation maps were computed for the two cerebral seed regions in each participant. The group-averaged, Fisher’s *r*-to-*z* transformed correlation map was generated for each seed region. As a result, brain areas with significant connectivity to the ROIs within each group were obtained with a *p* value <0.005 (uncorrected) at voxel level and a *p* value <0.05 with an extent threshold of 27 voxels for the clusters (AlphaSim correction).

In order to test for crossed laterality, direct comparisons of the left and right cerebral seed regions were computed via arithmetic subtraction of the *z* score correlation maps.

## Results

**Fig. 1:**
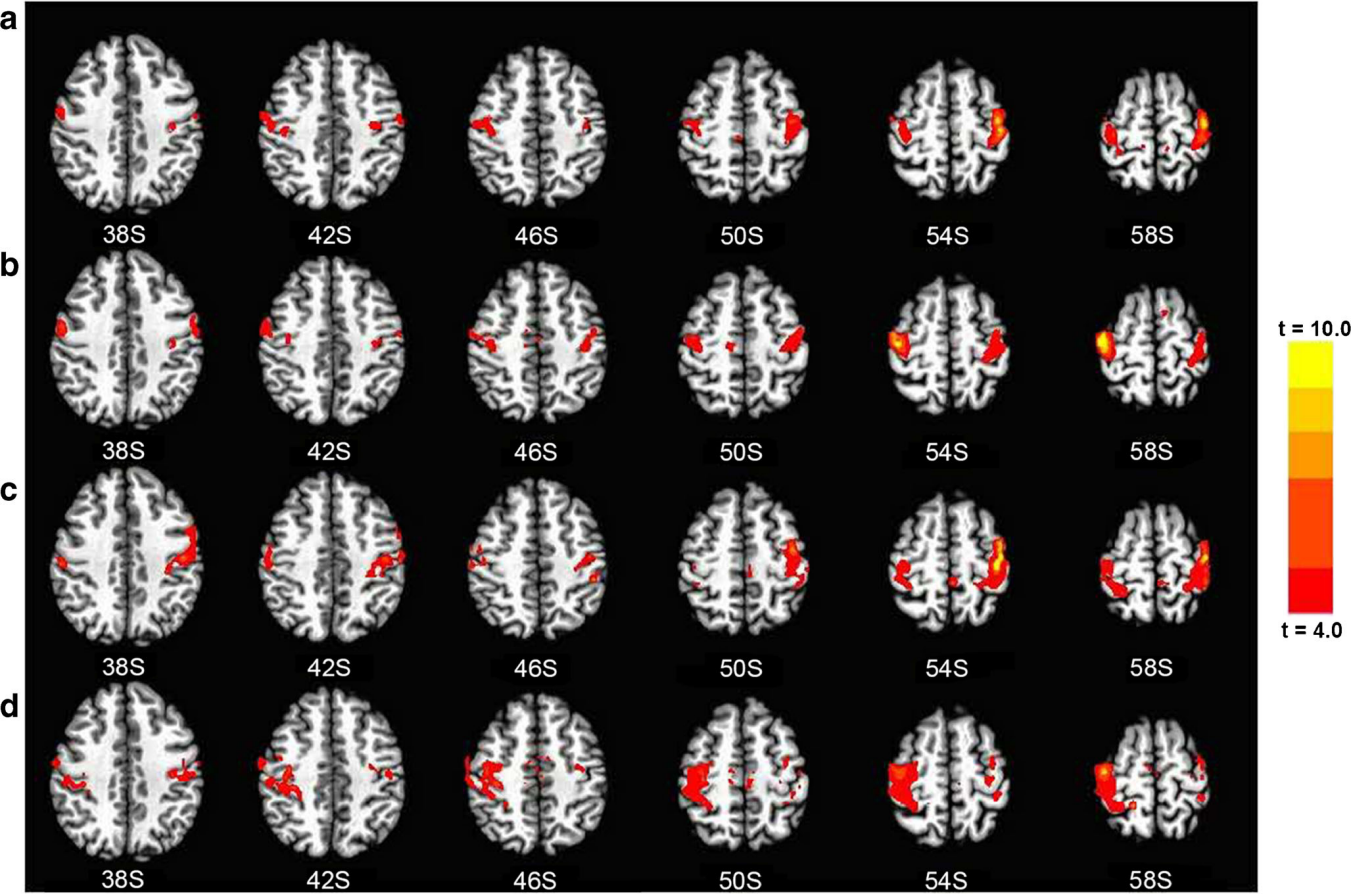
Functional connectivity map, healthy controls vs. patients. An ROI in the left-hand motor seed regions (*a*). An ROI in the right-hand motor seed regions (*b*). An ROI in the hand area contralateral to the injured side (*c*). An ROI in the hand area contralateral to the intact side (*d*). Cortical map showed significant functional connectivity between bilateral M1 areas and bilateral SMAs

In healthy volunteers, by placing an ROI in the left-hand or the right-hand motor seed regions, the cortical map showed significant functional connectivity between bilateral M1 areas and bilateral SMAs (Fig. 1 (a and b) and Table 2).

**Table 2:** Significant functional connectivity of healthy controls and patients

Group	ROIs	Regions	Talairach-Tournoux coordinates				
			Cluster size (mm^3^)	*x*	*y*	*z*	*t* value
Control	L_Hand	L_M1	8748	−40	−13	56	14.63
		R_M1	8532	30	−25	56	8.42
		SMA	1377	−7	−22	53	5.21
	R_Hand	L_M1	11,691	−43	−13	51	7.76
		R_M1	11,313	40	−20	56	10.21
		SMA	1269	13	−22	51	5.75
Patient	Injured side	L_M1	10,017	−40	−16	56	15.06
		R_M1	4860	26	−43	59	10.98
	Intact side	L_M1	4185	−25	−34	62	4.47
		R_M1	10,557	16	−43	59	10.98

*L* left, *R* right, *M1* the primary motor cortex, *SMA* supplementary motor area

By placing an ROI in the hand area contralateral to the injured side or the intact side, significant functional connectivity was found between bilateral M1 areas and bilateral SMAs (Fig. 1 (c and d) and Table 2).

**Fig. 2:**
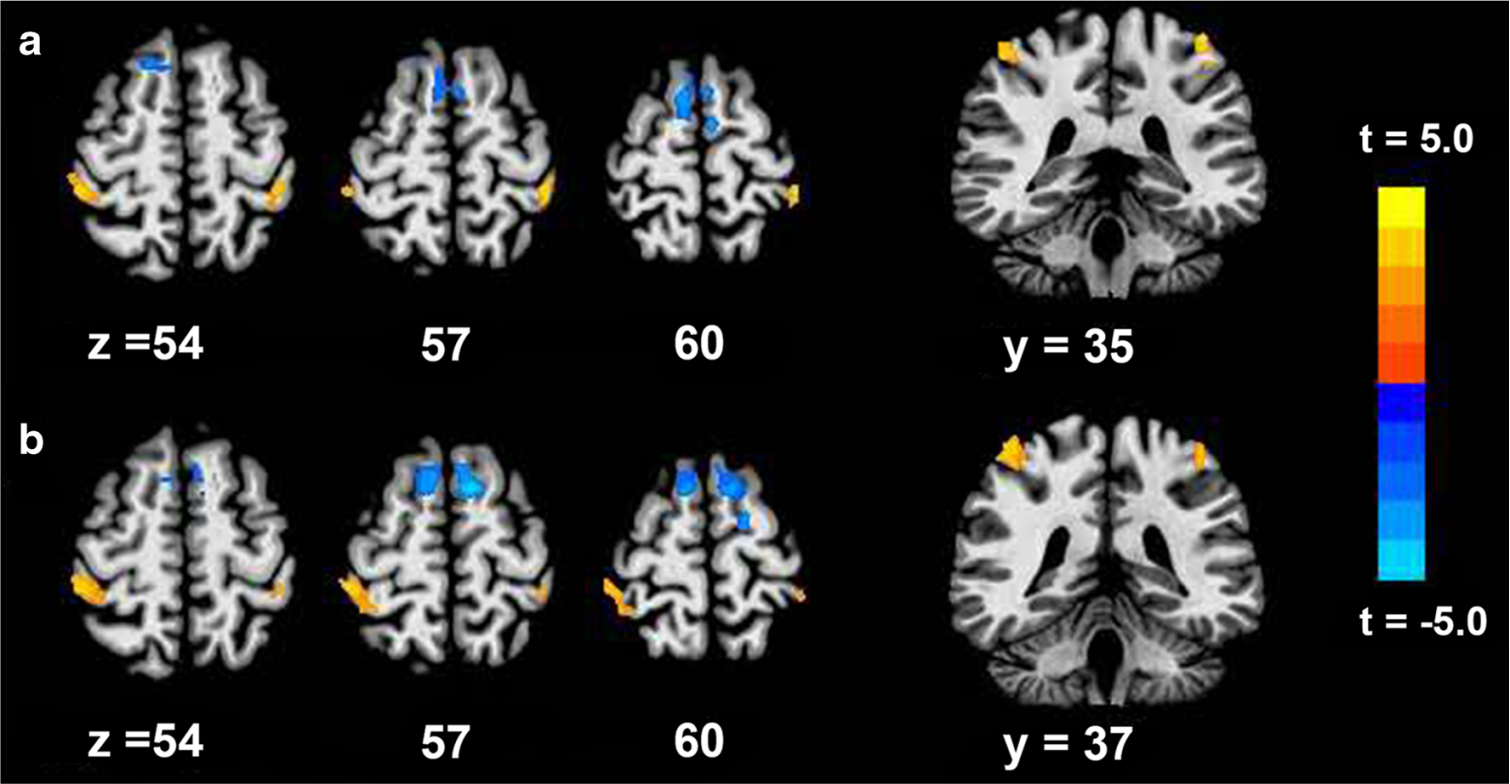
An ROI in the hand area contralateral to the injured side (*a*). An ROI in the hand area contralateral to the intact side (*b*). Compared with healthy volunteers, patients showed an increase in inter-hemispheric functional connectivity of the M1 areas in the resting-state fMRI and reduced functional connectivity between the two SMAs

Compared with healthy volunteers, the inter-hemispheric functional connectivity of the M1 areas in the resting-state fMRI was increased in the patients with BPAI after contralateral C7 nerve. Inter-hemispheric functional connectivity between the two SMAs was reduced in the patients (Fig. 2 (a and b) and Table 3).

**Table 3:** Significant differences of functional connectivity between healthy controls and patients

Contrasts	ROIs	Regions	Talairach-Tournoux coordinates				
			Cluster size (mm^3^)	*x*	*y*	*z*	*t* value
Patients > controls	Injured side	L_M1	1026	−40	−34	54	3.59
		R_M1	1053	26	−43	59	3.94
	Intact side	L_M1	1242	−40	−34	54	3.59
		R_M1	1809	43	−34	59	4.41
Patients < controls	Injured side	SMA	4266	−4	−7	62	4.97
	Intact side	SMA	2430	−7	7	56	4.65

*L* left, *R* right, *M1* the primary motor cortex, *SMA* supplementary motor area

## Discussion

As a non-invasive imaging modality, fMRI has revolutionized our understanding of functional networks and cerebral organization in both normal and pathological brains over the past several decades. Task-based functional imaging studies showed intra- and inter-hemispheric cortical plasticity in rats and humans following contralateral C7 nerve transfer for BPAI [6–10]. However, task-based fMRI scan is challenging for most BPAI patients who have to accomplish motor function of the affected wrist and fingers by bilateral associated movements, which result in movement artifacts. One of the important advantages of RS-fMRI is independence from functional tasks. In addition, the interactions of the motor networks can be investigated in the resting state after contralateral C7 nerve transfer.

In this study, alterations in the functional connectivity of motor cortex in BPAI following contralateral C7 nerve transfer were measured to investigate cortical reorganization. Our most important finding related to increased inter-hemispheric functional connectivity between the bilateral M1 areas in patients with BPAI after contralateral C7 nerve transfer compared with that of the controls. The strength of functional connectivity is consistent with the degree of spontaneous neuronal activity synchronization [11–13]. Functional connectivity reflects the intrinsic functional architecture of the human brain [11–13]. Changes in functional connectivity were found in subjects afflicted with central or peripheral nervous system disease [20–24]. Studies showed that resting-state inter-hemispheric functional connectivity and intrinsic horizontal functional connections of the primary motor areas were reduced following brachial plexus avulsion injury [21, 24]. In the present study, the motor function of the affected wrist and fingers in patients with BPAI after C7 nerve transfer recovered with contralateral movements. The findings in this study suggest that the increased inter-hemispheric functional connectivity was related to the increased synchronization of the two primary motor areas and changes in intrinsic functional architecture of the patient’s brain. This result suggests functional reorganization of the two primary motor areas in patients with BPAI after contralateral C7 nerve transfer.

The resting-state fMRI study in patients with BPAI after contralateral C7 nerve transfer was seldom reported, in contrast to task functional imaging studies. Accumulating evidence involving task functional imaging studies in patients with BPAI shows long-term plasticity between cortical hemispheres, in addition to cortical plasticity between neighboring regions in the same hemisphere [7, 25–27]. A study of long-term cortical remodeling in BPAI after contralateral C7 nerve transfer suggests that the motor control of the reinnervated limb was switched from the ipsilateral hemisphere of the affected plexus to the bilateral hemispheres and finally to the contralateral neural network activation [24], which is consistent with animal studies [9, 28]. The studies also suggest time-dependent cortical reorganization. In the current study, the affected wrist and fingers in patients after contralateral C7 nerve transfer moved with the contralateral movements, and increased synchronization of the two primary motor areas of the brain occurred at specific clinical stages. We believe that functional connectivity may be altered when patients controlled the injured limb independently. RS-fMRI can be used to further elucidate the mechanisms of functional recovery in patients with contralateral C7 nerve transfer.

In this study, reduced inter-hemispheric functional connectivity occurred between the two SMAs of patients. A study with resting-state functional connectivity in patients with BPAI also discovered decreased functional connectivity between the SMA and multiple brain regions [29]. The SMA is thought to be a key structure and played a higher role in behavioral planning and execution, such as alternate motor plans, task switching, acquisition of new motor skills, and motor selection [30–32]. The motor function recovery is initiated with the primary motor function, compared with the poor restoration of higher and complex motor function.

The present study has several limitations. First, the study is a cross-sectional investigation. Longitudinal investigation including different pre- and post-surgical time points is important to study the dynamics of cortical plasticity in patients with BPAI. In this study, including the normal controls, controls from pre-surgical BPAI patients might provide more information, because functional connectivity change might be from C7 nerve transfer and post-surgical functional exercise and might also have existed before C7 nerve transfer. Second, the sample size was small since the incidence of contralateral C7 nerve transfer for BPAI is rare. Further, the small size was not conducive to a study based on age groups, although it is known that younger subjects may display additional neural plasticity. A longitudinal study with a larger number of patients is needed to investigate the changes in functional connectivity. We also intend to comprehensively evaluate the functional imaging results combined with muscle strength grading.

In conclusion, this study demonstrates that the resting-state inter-hemispheric functional connectivity of the bilateral M1 areas was altered in patients with BPAI following contralateral C7 nerve transfer, suggesting functional reorganization of the cerebral cortex.

## Electronic supplementary material

Figure S1 (JPEG 66 kb)

High resolution image (TIFF 387 kb)
