# Supplementary figures and images for: Functional connectivity of motor cortical network in patients with brachial plexus avulsion injury after contralateral cervical nerve transfer: a resting-state fMRI study
Source: Neuroradiology. 2017 Feb 24;59(3):247–53. doi: 10.1007/s00234-017-1796-0 (PMC5371620; doi:10.1007/s00234-017-1796-0)

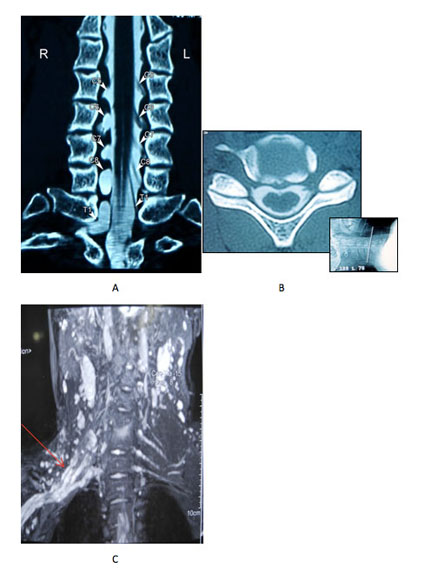

Supplement: Supplementary file 1 — (JPEG 66 kb) [file 234_2017_1796_Fig3_ESM.jpg]

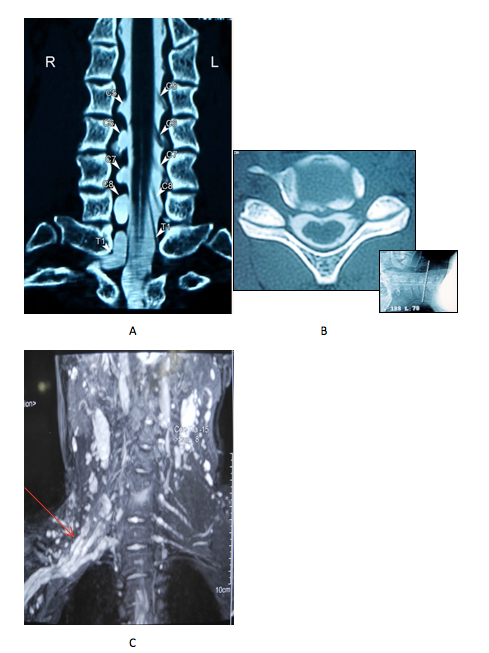

Supplement: Supplementary file 2 — High resolution image (TIFF 387 kb) [file 234_2017_1796_MOESM1_ESM.tiff]
